# The First Evidence of Gibberellic Acid’s Ability to Modulate Target Species’ Sensitivity to Honeysuckle (*Lonicera maackii*) Allelochemicals

**DOI:** 10.3390/plants12051014

**Published:** 2023-02-23

**Authors:** Csengele Éva Barta, Brian Colby Jenkins, Devon Shay Lindstrom, Alyka Kay Zahnd, Gyöngyi Székely

**Affiliations:** 1 Department of Biology, Missouri Western State University, 4525 Downs Drive, Agenstein-Remington Halls, St. Joseph, MO 64507, USA; 2 Hungarian Department of Biology and Ecology, Faculty of Biology and Geology, Babeș-Bolyai University, 5-7 Clinicilor St., 400006 Cluj-Napoca, Romania; 3 Institute for Research-Development-Innovation in Applied Natural Sciences, Babeș-Bolyai University, 30 Fântânele St., 400294 Cluj-Napoca, Romania; 4 Centre for Systems Biology, Biodiversity and Bioresources (3B), Babeș-Bolyai University, 5-7 Clinicilor St., 400006 Cluj-Napoca, Romania

**Keywords:** invasive plant species, allelopathy, phenolics, apigenin, luteolin, phytohormones, gibberellic acid (GA_3_), *Lonicera maackii*, *Brassica rapa*

## Abstract

Invasive species employ competitive strategies such as releasing allelopathic chemicals into the environment that negatively impact native species. Decomposing Amur honeysuckle (*Lonicera maackii*) leaves leach various allelopathic phenolics into the soil, decreasing the vigor of several native species. Notable differences in the net negative impacts of *L. maackii* metabolites on target species were argued to depend on soil properties, the microbiome, the proximity to the allelochemical source, the allelochemical concentration, or environmental conditions. This study is the first to address the role of target species’ metabolic properties in determining their net sensitivity to allelopathic inhibition by *L. maackii*. Gibberellic acid (GA_3_) is a critical regulator of seed germination and early development. We hypothesized that GA_3_ levels might affect the target sensitivity to allelopathic inhibitors and evaluated differences in the response of a standard (control, *Rbr*), a GA_3_-overproducing (*ein*), and a GA_3_-deficient (*ros*) *Brassica rapa* variety to *L. maackii* allelochemicals. Our results demonstrate that high GA_3_ concentrations substantially alleviate the inhibitory effects of *L. maackii* allelochemicals. A better understanding of the importance of target species’ metabolic properties in their responses to allelochemicals will contribute to developing novel invasive species control and biodiversity conservation protocols and may contribute to applications in agriculture.

## 1. Introduction

Invasive plant species are significant threats to biodiversity, negatively impacting native species, altering the community structure and dynamics, and affecting the ecosystem health and functions, regionally and globally [1,2,3,4]. A better understanding of the eco-physiological and molecular impacts that the invasives exert on native species and communities is essential for cost-effective and successful invasive species management, native species conservation, restoration, and agricultural weed management practices [5,6,7,8,9,10,11,12,13,14].

Amur honeysuckle (*Lonicera maackii* (Rupr.) Herder), a deciduous, highly successful invasive shrub, was introduced to the U.S. in the 18th century from Asia for its ornamental value [15,16]. In Asia, extracts from *Lonicera* spp. have traditionally been used as medicinal supplements to combat colds and influenza [17,18,19] and have been exploited for their anti-inflammatory [20,21], antioxidant, and anti-mutagenic properties [22,23,24].

*L. maackii* was a species recommended by the U.S. Soil Conservation Service for soil erosion control and habitat restoration. Its use for such applications likely contributed to the rapid spread and broad expansion of its habitat. Its invasive success was not recognized until the 1950s [15,25]. Today, *L. maackii* aggressively invades the deciduous forest-populated eastern and mid-North American regions. Infestations have been reported and cataloged from North Dakota to Texas in the South and in Massachusetts and Georgia in the North and Southeast [16,25,26]. The invasive success and widespread distribution of this species prompted intense scientific interest over the past decades, with studies revealing its complex, multitrophic impacts on native populations, communities, landscapes, and ecosystems. The complexity and varying impacts of *L. maackii* across ecosystems at multiple ecological scales make this species of high interest in serving as a model of invasion impacts [16]. The recent sequencing of the *L. maackii* genome [27] further emphasizes the increasing scientific interest in this species and the molecular traits underlying its high invasive success.

*L. maackii*’s widespread invasive ability is underpinned by a suite of invasive traits [28,29,30,31,32]. These include rapid growth and phenotypic plasticity [31,33,34,35,36,37,38,39], phenological differences from native species in the invaded habitat, resistance to environmental stresses [37,40,41,42], and long-range seed dispersal facilitated by birds, deer, or others [29,30,43,44,45]. *L. maackii* also synthesizes a variety of secondary metabolites, some of which act as herbivore deterrents or toxins [46,47,48,49,50].

Some of the metabolites synthesized in the roots, leaves, and fruits of *L. maackii* have also been reported to exert negative allelopathic activity, suppressing the germination, growth, survival, or reproduction of native plant species without auto-toxic impacts, possibly further enhancing *L. maackii’s* competitive ability [51,52,53,54,55,56,57]. Cipollini et al. (2008) [47] attributed these adverse effects to phenolic molecules and their derivatives isolated from *L. maackii* tissue extracts.

Studies found *L. maackii* fruit extracts to suppress or delay the germination of grass species (e.g., tall fescue, *Festuca arundinacea*, and Kentucky bluegrass, *Poa pratensis*), garden coreopsis (*Coreopsis lanceolata*), or a dwarf impatiens hybrid (*Impatiens walleriana*) [55]. On the other hand, leaf extracts only affected the latter negatively [55]. The negative impacts of *L. maackii* leaf extracts were also documented in woody species, with Trisel (1997) and Gorchov and Trisel (2003) showing a reduced germination of green ash (*Fraxinus pennsylvanica*) and sugar maple (*Acer saccharum*) [58,59]. The adverse allelopathic effects are not limited to *L. maackii*. Leaf extracts of another species, *L. japonica* (Japanese honeysuckle), reduced the growth of loblolly and shortleaf pine (*Pinus taeda* and *P. echinata*), an effect ascribed to the secondary phenolic metabolites identified from the leaf tissue [60]. Furthermore, *L. maackii* root and shoot extracts reduced the germination of several midwestern species’ seeds, such as jewelweed (*Impatiens capensis Meerb.*), tall thimbleweed (*Anemone virginiana* L.), and the model *Arabidopsis thaliana* [47,51,52,53,54,55,56,57]. Other studies reported no apparent allelopathic impacts of *L. maackii* on native herbaceous species [61]. Cipollini and Dorning (2008) also attributed confounding effects to *L. maackii* leaf extracts, reporting a decrease in the survival and a delay in the flowering of *A. thaliana*; however, this was associated with the increased seed production and leaf size at maturity [52]. These observations highlight a potentially complex phytochemical interaction between *L. maackii* and other species. It is also feasible for the described interactions between *L. maackii* and other species to be affected by the targets’ endogenous characteristics and, possibly, biotic or environmental factors, which may enhance or attenuate the allelopathic effects in a context-dependent manner [62]. Recent work also emphasizes that environmental pressures, such as stress conditions, may lower *L. maackii* performance; however, this is likely to be offset by the benefits gained in plant–plant competition by an increasing allelopathic potential [63].

Despite a good understanding of *L. maackii*’s impacts on ecosystems [6,16,30,33,52,59,61,64,65,66,67,68,69,70], the complex and potentially context-dependent direct chemical interactions between *L. maackii* and target plant species are not understood.

In the current work, we sought to evaluate the potentially fundamental role of the seed germination stimulator gibberellic acid (GA_3_) [71,72,73,74,75,76,77,78,79] in determining target sensitivity to *L. maackii* allelochemicals. We used a model system that allowed for the comparative evaluation of the impacts of *L. maackii* leaf metabolites on the germination and growth of control, GA_3_-overproducing, and GA_3_-deficient *Brassica rapa* varieties [80,81,82].

A better understanding of the physiological and biochemical controls modulating the sensitivity of target species to allelopathic influences is expected to contribute to developing innovative invasive species management approaches and novel applications in agriculture and weed management under current and future climatic conditions.

## 2. Results

### 2.1. Lonicera maackii Leaf Extracts Inhibit Brassica rapa Seed Germination

**Figure 1:**
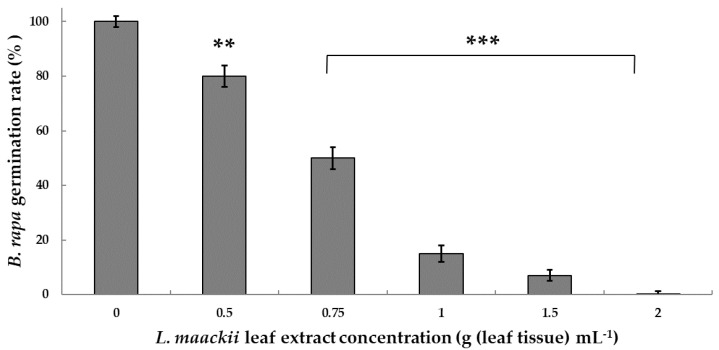
The impact of *Lonicera maackii* leaf extracts on *Brassica rapa* seed germination. Standard, *Rbr* seeds were treated with *L. maackii* aqueous leaf extracts for 24 h. The extract concentrations ranged from 0 to 2 g (leaf tissue) mL^−1^. Bars represent mean values ± SD (n = 60, N = 3). Significantly different means, compared to untreated controls, according to Student’s *t*-test, are marked with ** *p* = 0.01–0.001 and *** *p* < 0.001.

*L. maackii* extracts, prepared from late-season harvested leaves, significantly decreased *B. rapa* seed germination rates in a range from 20 to 95% compared to controls (Standard variety, *Rbr*, Wisconsin Fast Plants^®^) in Petri dish assays in a concentration range of 0.5–2 g (leaf tissue) mL^−1^. The effects were concentration-dependent, with lower germination rates associated with higher concentrations of the applied extract. All control *B. rapa* seeds, treated with sterile distilled water, germinated within 24 h. The germination rates of seeds treated with 0.5, 0.75, 1, and 1.5 g (leaf tissue) mL^−1^ *L. maackii* extracts were reduced by 20, 50, 85, and 95%, respectively. Treatments with 2 g (leaf tissue) mL^−1^ extracts fully inhibited *B. rapa* seed germination (Figure 1).

**Figure 2:**
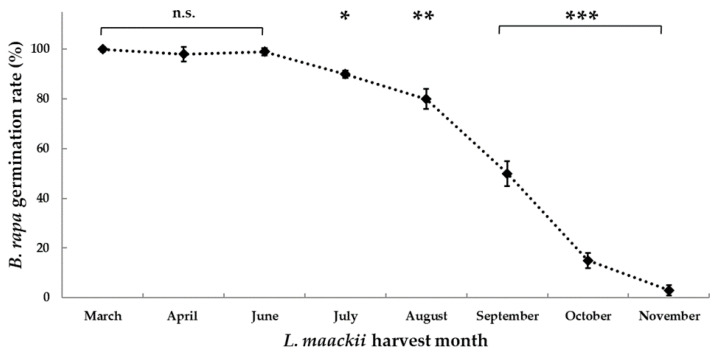
Seasonal variations in the impact of *Lonicera maackii* leaf extracts on *Brassica rapa* seed germination. *Rbr* Standard *B. rapa* seeds were treated for 24 h with 1 g (leaf tissue) mL^−1^ *L. maackii* extracts prepared from leaves regularly harvested between March and November 2021. Germination rates were compared to *Rbr* germination under control conditions when treated with sterile water. Control germination was 100% after 24 h. Diamonds represent mean values ± SD (n = 60, N = 3). Significantly different means, with differences compared to controls, according to Student’s *t*-test, are marked with * *p* = 0.05–0.01, ** *p* = 0.01–0.001, and *** *p* < 0.001; n.s. = not significant.

*L. maackii* extracts of a 1 g (leaf tissue) mL^−1^ concentration, prepared from mature leaves in different moments of the season, from early spring to late fall, had varying effects on *B. rapa* var. *Rbr* germination. Early-season leaves (harvested between March and June) did not inhibit the germination of *B. rapa* seeds. Leaves harvested later, from mid-summer to late fall, had a progressively increasing negative impact on *Rbr* seed germination. Germination rates declined by 10, 20, 50, 85, and 95% in response to *L. maackii* extracts prepared from leaves harvested monthly between July and November, respectively (Figure 2).

### 2.2. Gibberellic Acid Influences Brassica rapa’s Sensitivity to Lonicera maackii Allelopathic Effects

**Figure 3:**
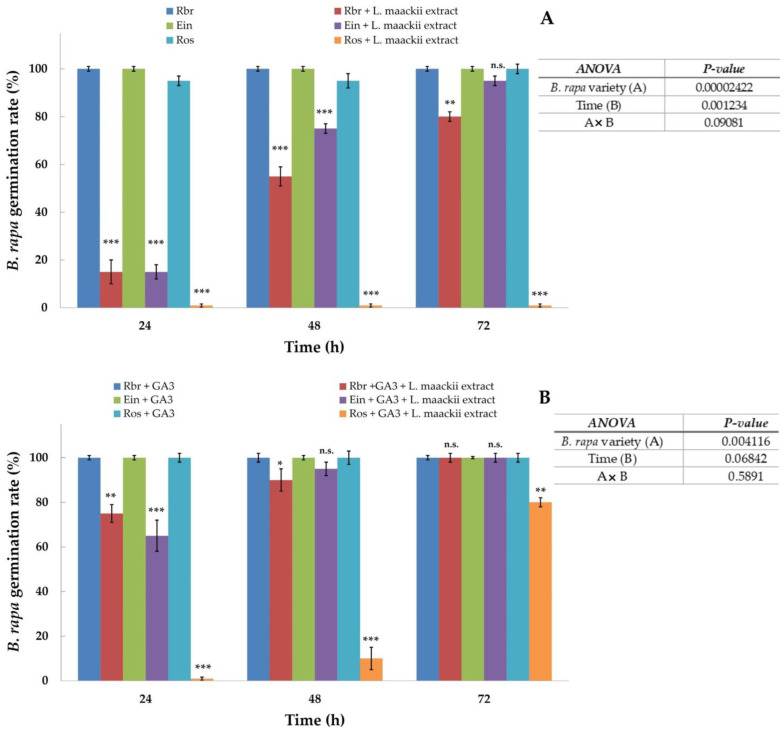
The impact of endo- and exogenous gibberellin on *Brassica rapa* seed germination exposed to *Lonicera maackii* leaf extracts. Standard (*Rbr*), gibberellin-overproducing (*ein*), and gibberellin-deficient (*ros*) *B. rapa* seeds were treated with 1 g (leaf tissue) mL^−1^ *L. maackii* extracts without (panel **A**) or in the presence of supplemental exogenous GA_3_ of 100 µmol (panel **B**) for 72 h. Corresponding control treatments included *Rbr*, *ein*, and *ros* seeds imbibed with sterile distilled water (panel **A**) or sterile distilled water supplemented with 100 µmol GA_3_ (panel **B**). Bars represent means ± SD (n = 60, N = 3). Significantly different means, compared to control treatments for each variety in each group, according to Student’s *t*-test, are marked with * *p* = 0.05–0.01, ** *p* = 0.01–0.001, and *** *p* < 0.001; n.s. = not significant. Two-way ANOVA was used to test for differences in the germination of the used varieties treated with *L. maackii* extracts without (panel **A**) or in the presence (panel **B**) of exogenous GA_3_ over time. *p*-values for variety responses and their compared changes over time are shown as table insets on (panel **A**) (for treatments in the absence of exogenous GA_3_) and (panel **B**) (for treatments in the presence of exogenous GA_3_).

**Figure 4:**
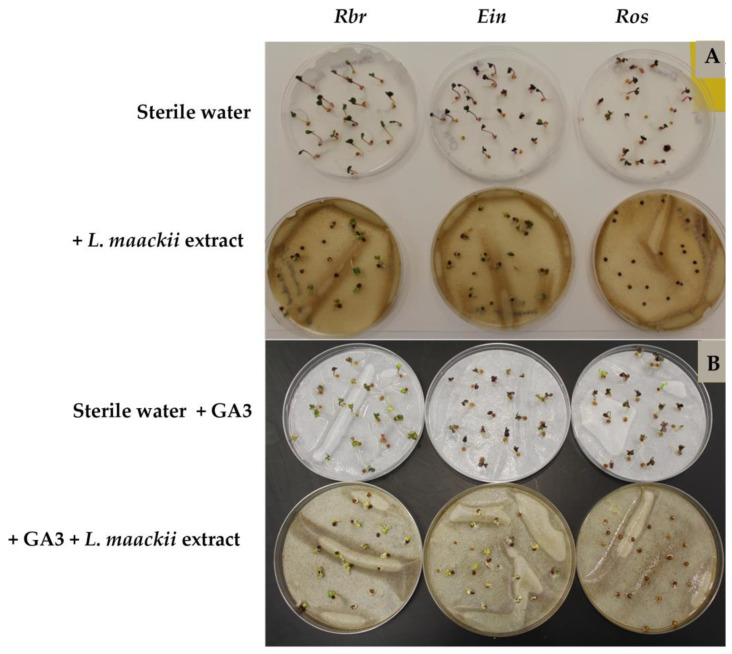
Representative Petri dish seed assay plates showing the impact of endo- and exogenous gibberellin on *Brassica rapa* seed germination exposed to *Lonicera maackii* leaf extracts. Standard (*Rbr*), gibberellin-overproducing (*ein*), and gibberellin-deficient (*ros*) *B. rapa* seeds were treated with 1 g (leaf tissue) mL^−1^ *L. maackii* extract without (panel **A**) or in the presence of 100 µmol exogenous GA_3_ (panel **B**) for 72 h. Corresponding control treatments included *Rbr*, *ein*, and *ros* seeds imbibed with sterile distilled water (panel **A**) or sterile distilled water supplemented with 100 µmol GA_3_ (panel **B**).

The germination success in seeds treated with *L. maackii* extracts varied substantially in the function of the endogenous gibberellin production capacity of the tested *B. rapa* varieties. Significant differences were observed between the response of the GA_3_-overproducing, tall (*ein*, elongated internode) variant, the gibberellin-deficient, rosette-dwarf (*ros*) *B. rapa* variant, and the standard, *Rbr* variety, producing physiological levels of gibberellins (Figure 3A and Figure 4A). The control seeds of all three varieties, treated with sterile water, germinated within 72 h after imbibition, with the seeds of the *Rbr* and *ein* varieties germinating after 24 h. The *ros* variety control germination rate was 92, 95, and 100% after 24, 48, and 72 h, respectively. After twenty-four hours of imbibition with 1 g (leaf tissue) mL^−1^ *L. maackii* extracts prepared from leaves harvested in October, *Rbr* and *ein* seed germination significantly decreased in both varieties by over 85%. After 48 h, 55% of the *L. maackii*-treated *Rbr* and 75% of the *ein* seeds germinated, while after 72 h, 80% of the *Rbr* and 95% of the *ein* seeds germinated. Imbibition with the *L. maackii* leaf extract completely inhibited the germination of the gibberellin-deficient *ros* seeds; no seedlings germinated within 72 h. The statistical analysis revealed a significant impact of the applied *L. maackii* leaf extract on the germination success of all varieties when compared to the corresponding controls; this effect was particularly enhanced at the beginning of the treatment after 24 and 48 h. While *L. maackii* leaf extracts negatively affected the germination success of all varieties, delaying the germination of both *Rbr* and *ein* varieties, *ein* seeds showed a lower sensitivity to *L. maackii*-derived inhibitors than *Rbr*. In contrast, the germination of the *ros* seeds was fully inhibited by the *L. maackii* leaf extract (Figure 3A and Figure 4A). Further statistical evaluation of these data with two-way ANOVA analysis of variance also supports that the variety responses to *L. maackii* allelochemicals are significantly different. However, these differences become less accentuated over time (Figure 3A inset), except for the *ros* variety, whose seeds did not germinate within our observation window.

The response of *B. rapa* varieties revealed a similar trend when *L. maackii* extracts were administered in conjunction with a supplemental exogenous 100 µmol GA_3_ solution (Figure 3B and Figure 4B). The controls of all three varieties, treated with sterile water supplemented with exogenous GA_3_, germinated after 24 h, including the GA_3_-deficient dwarf variety. After 24 h, 75 and 65% of *Rbr* and *ein* seeds imbibed with the combination of GA_3_ + *L. maackii* leaf extracts germinated. Supplementation with exogenous GA_3_ enhanced the germination rates of *L. maackii*-treated *Rbr* and *ein* seeds and, after 48 h, *ros* seeds as well, as compared to the rates recorded without the supplement (Figure 3A and Figure 4A). Exogenous GA_3_ enhanced the germination of *Rbr* and *ein* seeds treated with the *L. maackii* extract after 24 h (Figure 3B), as compared to the *Rbr* and *ein* seed germination rates without exogenous GA_3_ (Figure 3A). At later timepoints, a further stimulation of *Rbr* and *ein* was detected in the presence of exogenous GA_3_ (Figure 3A,B). After 48 h, 90 and 95% of *Rbr* and *ein* seeds germinated, respectively. The complete germination of the *Rbr* and *ein* seeds treated with GA_3_ + *L. maackii* leaf extract was recorded by the 72 h timepoint.

Finally, in the presence of exogenous GA_3,_ the seeds of the rosette-dwarf, gibberellin-deficient variety were also able to partially overcome the harmful effects of *L. maackii* inhibitors, and, albeit with a delay compared to the other varieties and controls, 80% of the *ros* seeds germinated within 72 h. Two-way ANOVA analysis of variance revealed that the variety dependence of the response of *B. rapa* seeds to *L. maackii* allelochemicals is still significant in the presence of exogenous GA_3_; however, the differences are attenuated on a longer timescale (Figure 3B and inset).

**Figure 5:**
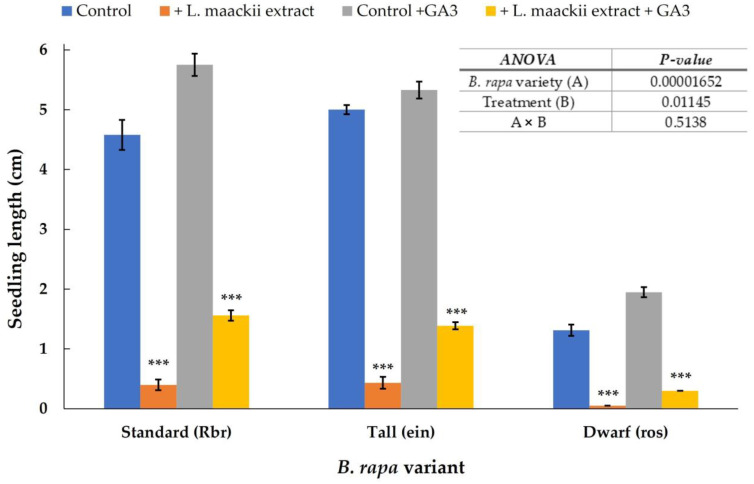
The impact of endo- and exogenous gibberellin on *Lonicera maackii* leaf extract-treated *Rbr*, *ein*, and *ros Brassica rapa* seedling growth. Standard (*Rbr*), gibberellin-overproducing (*ein*), and gibberellin-deficient (*ros*) *B. rapa* seeds were treated with 1 g (leaf tissue) mL^−1^ *L. maackii* extract without (orange bars) or in the presence of 100 µmol exogenous GA_3_ (yellow bars) for 72 h. Corresponding control treatments included *Rbr*, ein, and *ros* seeds imbibed with sterile distilled water (blue bars) or sterile distilled water supplemented with 100 µmol GA_3_ (grey bars). Bars represent means ± SD (n = 60, N = 3). Significantly different means, compared to control treatments for each variety in each group, according to Student’s *t*-test, are marked with *** *p* < 0.001. Two-way ANOVA was used to test for differences in the growth of control and treated *B. rapa* varieties in the function of endo- and exogenous GA_3_ treated with *L. maackii* extracts. *p*-values for variety, treatment, and variety x treatment responses are shown in the table inset.

Within the first 72 h after exposure to *L. maackii* leaf extracts, we detected significant differences in the growth and development of seedlings with differing capacities to produce GA_3_. Similar differences were recorded when seedlings were treated with exogenous GA_3_ in conjunction with the *L. maackii* extract (Figure 5). The growth pattern of the control *Rbr*, *ein*, and *ros* varieties, treated with sterile water, showed the typical response expected from varieties synthesizing physiological amounts of GA_3_, overproducing and showing deficiencies in GA_3_ synthesis. The *ein* variety seedlings reached 5 cm in length, the *Rbr* seedlings reached 4.5 cm, and the *ros* seedlings reached 1.3 cm. Imbibition with *L. maackii* extracts significantly suppressed the growth of both *Rbr* and *ein* varieties, with the average seedling length not exceeding 0.5 cm for either variety. *Ros* seedlings did not germinate within the timeframe of our observation window; therefore, their length was considered 0 cm.

Exogenous GA_3_ supplementation benefitted, in particular, the *Rbr* and *ros* seedlings under control conditions in sterile water supplemented with GA_3_. The average seedling length was 5.7, 5.45, and 1.95 cm in the *Rbr*, *ein*, and *ros* varieties. While the exogenous GA_3_ did not fully compensate for the inhibitory effects of *L. maackii* leaf extracts, the seedling lengths recorded 72 h after the exposure to the combination of *L. maackii* extract + GA_3_ were more extensive for each variety compared to those of the same variety exposed to *L. maackii* extracts alone. *Rbr*, *ein*, and *ros* seedlings reached an average length of 1.5, 1.4, and 0.4 cm when treated with *L. maackii*, extracts supplemented with exogenous GA_3_. Two-way ANOVA was used to test for differences in the growth of control and *L. maackii* extract-treated (with or without exogenous GA_3_) *B. rapa* varieties. Analyses revealed a significant, variety-dependent growth response to the *L. maackii* leaf extract treatment and supported significant differences between the control and *L. maackii* extract-treated samples both in the presence and absence of exogenous GA_3_ (Figure 5 inset).

### 2.3. Apigenin, Luteolin, and Their Combination Exert Varying Effects on the Germination and Growth of B. rapa Seeds

**Figure 6:**
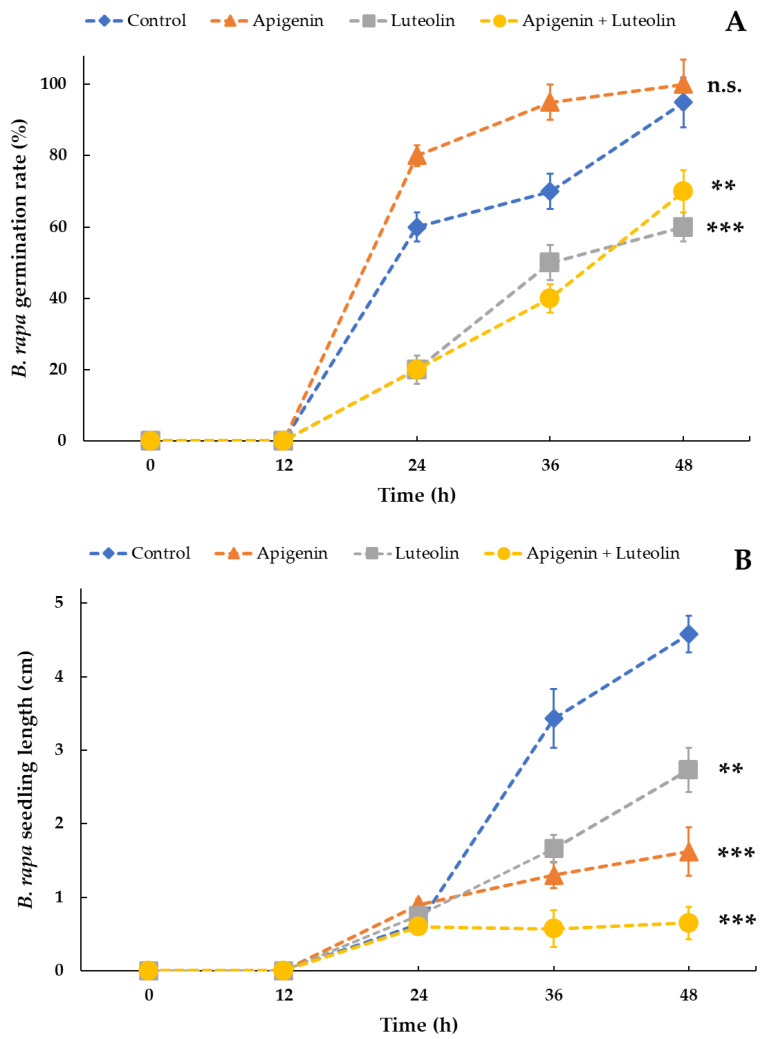
The impact of API, LUT, and their combination on the germination (panel **A**) and seedling growth (panel **B**) of *Rbr* (Standard) *Brassica rapa* seeds and seedlings. Seeds were treated with 50 µg mL^−1^ solutions of API (orange triangles), LUT (grey squares), and the combination of API + LUT (yellow circles) in 0.05% DMSO for 48 h in Petri-dish assays. Control seeds were treated with an aqueous solution of 0.05% DMSO. Markers represent mean values ± SD (n = 60, N = 3). Significantly different means, with differences compared to controls, according to Student’s t-test, are marked with ** *p* = 0.01–0.001 and *** *p* < 0.001. n.s. = not significant.

**Figure 7:**
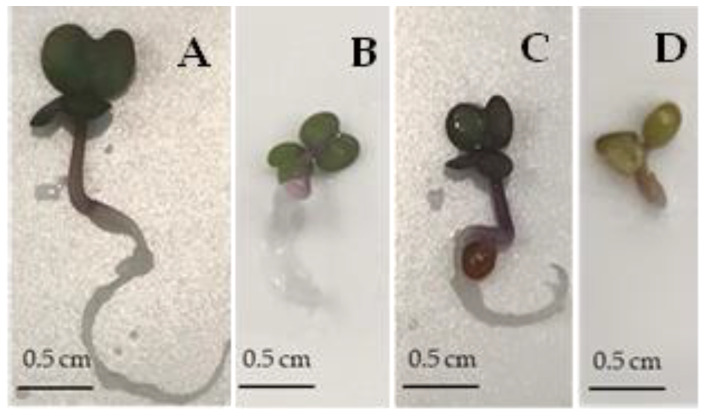
The impact of API, LUT, and their combination on *Rbr* (Standard) *Brassica rapa* seedling development. The figure shows representative example photographs of seedlings developed from seeds treated with 50 µg mL^−1^ solution of API (**B**), LUT (**C**), and the combination of API + LUT (**D**) in 0.05% DMSO for 48 h. Control seeds (**A**) were treated with an aqueous solution of 0.05% DMSO.

Apigenin (API) and luteolin (LUT) have been previously identified from aqueous extracts of *L. maackii* leaves as dominant flavones with potential allelochemical properties [47]. In seed assays, 50 µg mL^−1^ solutions of API, LUT, and their combination triggered various responses in the germination and development of *B. rapa* var. *Rbr* (Figure 6 and Figure 7). The allelochemical concentration for this test was selected to represent concentrations likely occurring under field conditions due to *L. maackii* leaf decomposition at the end of the growing season in the soil [47]. API stimulated the germination of *Rbr* seeds, especially in the early stages of the treatment, with higher germination rates observed, particularly after 24 and 36 h, when compared to the controls. After 48 h, this effect was lost, with all of the control and API-treated seeds germinating.

On the other hand, API had a significant suppressive impact on the seedlings’ growth compared to untreated controls. The average API-treated seedling length was 1.5 cm compared to untreated controls, whose average size reached 4.7 cm within 48 h. LUT suppressed germination and growth, although its effects on growth were weaker than those of API. Only about 60% of LUT-treated seeds germinated after 48 h, and the seedling size averaged 2.8 cm. The combination of API + LUT had a strong inhibitory effect on germination, similar to that of LUT, inhibiting germination by 35%.

Moreover, it had a highly significant suppressive impact on growth compared to API or LUT alone. The average seedling lengths did not exceed 0.7 cm when treated with API + LUT. The representative photographs of seedlings in Figure 7 also indicate that while API, LUT, and their combination suppress seedling growth, combining the two flavones affects root and hypocotyl development the strongest.

## 3. Discussion

In the Midwestern U.S., *L. maackii* has been recognized to have an aggressive invasive success and devastating multitrophic impacts on ecosystems, rapidly invading forests and disturbed landscapes across N. America [15,25,26]. Its invasive success relies on several competitive mechanisms, which make this species a good candidate for an invasive model [16]. One of the numerous strategies employed by invasive plant species is the synthesis of secondary metabolites with allelopathic properties. When released into the environment, these chemicals may influence their neighbor’s germination, growth, development, reproduction, and overall survival [8,83,84,85,86,87,88,89,90,91,92]. Allelopathic invasives, including *L. maackii*, have raised numerous concerns for species conservation and restoration, with further challenges posed by a warming climate. Climate change is likely to enhance the competitive relevance of allelochemistry in the biological invasion strategies of this species [63]. Therefore, a better understanding of these interactions is pivotal for biodiversity conservation, landscape and weed management, as well as potential applications in agriculture and forestry.

*Lonicera* spp. synthesize and release various bioactive phytochemicals from their live or degrading root, shoot, leaf, and fruit tissues [16,17,51,52,53,54,55,56,57,60,61,62]. These metabolites may play essential roles in plant defense against abiotic stressors, such as high-intensity and ultraviolet radiation [47,51], and protection against herbivores [46,47,48,49,50]. It is likely that their early recognized medicinal properties may also be attributed to their specialized phytochemistry [17,18,19,20,21,22,23,24]. Nevertheless, *L. maackii* phytochemicals may also be released into the soil through roots or leaf litter decomposition, negatively affecting the germination, growth, and development of native species [47,51].

Notably, most studies evaluated the phytochemical composition and allelopathic impacts of *L. maackii* in extracts prepared in organic solvents [47,85,93], with few exceptions [51,54,55,56,94]. However, in realistic field settings, the metabolites that are most likely effective in inhibiting other plant species must be water-soluble. In our study, we used a passive extraction method mimicking the progressive, gradual leaching of water-soluble phytochemicals from dense *L. maackii* leaf litter into the soil water at the end of its growing season. Leachates derived from late-season leaf litter inhibited the germination of *B. rapa* seeds in a concentration-dependent manner, with the more concentrated extracts exerting the highest inhibitory effects and a 2 g (leaf tissue) mL^−1^ concentration being lethal to the tested seeds (Figure 1). Even though the net impact of allelochemical products may also be affected by other factors [53,54,95,96,97,98,99,100,101], possibly attenuating their effects, they may substantially alter germination success and plant development [47,51,52,53,54,55,56,57,94]. Concentrations similar to those used in this study were detected in the soil for various phenolics [99,100,101,102].

These data are in good agreement with earlier descriptions of the inhibitory impacts of *L. maackii* leaf extracts on a variety of other species, including *Arabidopsis* spp., green ash, sugar maple, or an impatiens hybrid [47,51,52,53,54,55,56,57,58,59,60,61], and provide additional support for the *L. maackii* leaf litter inhibitory effects on yet another species. We chose *B. rapa*, a member of the Brassicaceae family, as the model species for our studies, as its wild varieties are widespread across the U.S., including areas affected by *L. maackii* infestations [26]. Moreover, *B*. *rapa* is sensitive to water-soluble allelochemicals released from various other allelopathic species [103,104,105,106,107,108,109].

Ontogeny and seasonal variations in environmental conditions control the synthesis of constitutive secondary metabolites in plant tissues [110,111,112,113,114,115,116,117,118,119,120,121,122,123,124,125,126,127]. Age-dependent and seasonal variations in the synthesis of terpenoids, phenolics, and alkaloids in leaf tissue have been commonly described and frequently attributed to, among other factors, the availability of metabolic precursors for their synthesis, deriving from primary metabolism [115,116,117,118,120,121,122,123,124,125,126,127]. The duration of *L. maackii’s* life cycle and period of photosynthetic competency exceeds that of the native vegetation in the invaded regions. *L. maackii* defoliation was recorded very late in the fall, sometimes retaining leaves until early in the following spring [16,40]. We hypothesized that leaf ontogeny also affects the synthesis and accumulation of metabolites with allelopathic properties in *L. maackii*. Extracts prepared from *L. maackii* leaves at non-lethal concentrations (1 g (leaf tissue) mL^−1^, Figure 1) inhibited *B. rapa* germination most significantly, and increasingly, only when prepared from tissue harvested in the late season, from September to November (Figure 2). Such seasonal variation in the inhibitory potential of *L. maackii* leaf extracts was not unexpected. Our data also support an earlier assessment provided by Cipollini and coworkers on specific variations in the synthesis of some phenolic metabolites in *L. maackii* leaves in summer versus fall months [47]. Some of those metabolites are also hypothesized to be responsible for the species’ allelopathic impacts. Like most other secondary metabolites, phenolics are versatile molecules and play various roles in plants [47]. Cipollini and coworkers ascribed the differential accumulation of select phenolics from among the 13 metabolites identified from *L. maackii* leaf extracts to their potentially varied functions [47]. They suggested that synthesizing phenolics that protect leaves from ultraviolet radiation-induced damages may peak during the summer months. Other metabolites, which are deterrents for herbivore activity, may be synthesized to best fit the herbivore activity cycle during each season [47].

Nevertheless, Cipollini and coworkers [47] did not address the net inhibitory potential of whole aqueous leaf extracts, as the current work does. One of the novelties in our results is the evidence that the strength of *L. maackii* leaf extracts in inhibiting *B. rapa* germination increases during the fall. We suggest the progressive inhibitory capacity of seasonal *L. maackii* extracts to be a proxy for the variations in the biosynthesis of allelopathic inhibitors in this species’ leaves. It is also likely that leaf litter forming in the fall breaks down during the wet season, leaching into soil water, with expected inhibitory effects exerted in early spring, when conditions become favorable for seed germination.

Today, allelochemicals are frequently argued to serve as “novel chemical weapons” against naïve native species that have not yet been exposed to them. Consequently, native species may be expected to have a higher sensitivity to allelochemical inhibition than species cohabiting the environment from which the invasive originated [8,83,128]. Several metabolic candidates were isolated from *Lonicera* spp., such as phenolics, flavones, flavonoids, their glucoside derivatives, phenolic acids, and iridoids as likely allelopathic inhibitors [47,58,59,60,129]. Nevertheless, the net allelopathic impacts varied substantially in studies using different plant species and variable controlled or field conditions [47,51,52,53,54,55,56,57,58,59,60,61,62]. When considering the context-dependency of allelopathic effects, under field conditions, soil properties, the microbiome, the proximity to the allelochemical source, or other environmental conditions were suggested to affect the net negative impact of *L. maackii* metabolites on target species [16,51,52,62,98,99,130].

Surprisingly, however, to date, no study has considered the possibility that the confounding effects reported earlier regarding *L. maackii*’s net impact on different species might also be influenced by endogenous recipient-dependent factors.

Allelochemicals affect various physiological and metabolic processes in plants. These include alterations in the cellular ultrastructure; the inhibition of cell division and elongation; imbalances in the antioxidant system; increases in cell membrane permeability; and alterations in enzyme activities, respiration, photosynthesis, water and nutrient uptakes, and plant growth regulation (reviewed by [89]). A target species’ sensitivity to allelochemical-induced effects may also depend on their metabolic properties. Phenolics and their derivatives are some of plant tissues’ most prevalent and extensively studied allelochemical groups [131,132,133,134,135,136,137]. Phenolics were also suggested to be the predominant allelochemical molecules in *L. maackii* leaves [16,47,58,59,60,138].

Substantial evidence supports that some plant species can counteract allelopathic phenolics through direct counter-allelopathic defense or other metabolic strategies. For example, Weir and coworkers showed that oxalate synthesized and excreted by *Gaillardia grandiflora* and *Lupinus sericeus* could neutralize catechin, an allelopathic substance released by *Centaurea maculosa* [139]. Exogenous oxalate could also protect *Arabidopsis* spp. from catechin-induced allelopathy [139]. Species such as *Lolium perenne*, *Trifolium repens*, *Rumex aquaticus*, and others were stimulated by concentrated allelochemical solutions, making use of them as fertilizers [84,85,140,141]. Legumes and species with larger seeds are speculated to be more likely to resist allelopathy due to their abundant nutrition and energy, which can be invested into resistance [142]. Garlic root allelochemicals altered the tomato seedling transcriptome, oxidant–antioxidant, and growth-regulating phytohormone balance, inducing resistance [143].

Phytohormones are critical regulators of plants’ vegetative and reproductive cycles. Environmental factors affect the transition from dormancy to germination competency in seeds. However, germination is tightly regulated by the balance between two phytohormones. Abscisic acid (ABA) sustains dormancy, while gibberellic acid (GA_3_) is required in high amounts, along with low ABA/GA_3_ ratios, to alleviate dormancy in order to permit germination [71,73,79]. GA_3_ stimulates seed germination, enhancing imbibition, weakening the seed coat, and activating hydrolytic enzymes. Germination involves bidirectional interactions between the embryo and the endosperm. The endosperm senses the appropriate conditions for germination, hence regulating the embryo’s growth. The embryo controls amylase release and endosperm degradation. Amylases break down the storage nutrients required for the nutrition and development of the embryo [72,73,74,75,76,77,78,79]. GA_3_ also stimulates the expression of genes responsible for embryonic cell expansion in the early stages of germination and seedling development [77].

The biosynthesis of GA_3_ was previously shown to be significantly inhibited by phenolic allelochemicals [137,143]. Cheng and coworkers also suggested that the allelochemical-induced alteration in phytohormone levels might increase plant resistance [143].

Here, we demonstrated that a standard (*Rbr*) *B. rapa* variety is sensitive to *L. maackii* aqueous extracts, supporting earlier sensitivity studies by Cipollini and colleagues [47]. These extracts inhibited *B. rapa* seed germination in a concentration-dependent manner (Figure 1), also controlled by *L. maackii* leaf ontogeny (Figure 2). In addition to the standard variety, using other *B. rapa* varieties with capacities to synthesize GA_3_ differing from that of the *Rbr*, we also tested the hypothesis that elevated endogenous GA_3_ levels may provide resistance against *L. maackii* allelochemicals.

We assessed the germination success and early-stage growth and development of a GA_3_-overproducing (*ein*) and a GA_3_-deficient (*ros*) *B. rapa* variety [81,82] treated with concentrations of *L. maackii* leaf extracts that are non-lethal to the *Rbr* variety (1 g (leaf tissue) mL^−1^, Figure 1). Compared to the sensitivity of the *Rbr*, seeds of the *ein* variety were less sensitive to inhibition. At the same time, *ros* proved to be the most inhibited by *L. maackii* allelopathic metabolites, with no germination recorded up to 72 h after treatment. By that time, most seeds of the other varieties and all of the water-treated controls germinated (Figure 3A and Figure 4A). In addition, exogenous GA_3_, in physiologically relevant concentrations (100 µmol, [144]), further decreased the sensitivity of all three tested *B. rapa* varieties to *L. maackii* inhibitors. The exogenous GA_3_ supplement was sufficient to substantially compensate for the diminished endogenous GA_3_ synthesis in the *ros* variety, with 78% of *ros* seeds exposed to *L. maackii* allelochemicals germinating within 72 h (Figure 3B and Figure 4B). We also noted a similar trend in the early seedling growth, with both elevated endo- and exogenous GA_3_ compensating for the inhibitory effects of *L. maackii* extracts (Figure 5). The highly significant differences in the sensitivity of *B. rapa* varieties and the impact of exogenous hormone supplements (Figure 3 and Figure 5, and insets) suggest that GA_3_ plays a substantial role in determining a species’ sensitivity and response to *L. maackii* allelochemicals. The observed differences between the growth and development of seedlings could be a consequence of the direct effects of GA_3_ on seed germination, and not a direct effect of GA_3_ on growth. We stipulate that the earlier-reported differences in the sensitivity of various species to *L. maackii* allelopathic inhibition [47,51,52,53,54,55,56,57,58,59,61,62] may, at least in part, be attributable to potential differences in the GA_3_ synthetic ability of the species. However, further studies are needed to ascertain these differences in various native species cohabiting with *L. maackii* to identify candidates with the highest capacity for resistance.

To our knowledge, the current work is the first to examine the role of a target species’ metabolic properties in determining their net sensitivity to allelopathic inhibition by *L. maackii*.

Earlier, Cipollini and coworkers [47] identified 13 phenolic metabolites from degrading *L. maackii* leaves. They suggested that API, LUT, and their glycosidic derivatives are likely allelopathic candidates. Flavones and flavonoids are phenolic metabolites commonly synthesized by many plant species, with demonstrated bioactivity [145]. The molecules isolated from *L. maackii* by Cipollini and colleagues [47] were also detected in other species such as *Cistus ladanifer* and *Matricaria chamomilla*. These species are also allelopathic [102,110,146]. From the metabolites analyzed by Cipollini and coworkers [47], we tested the effects of API, LUT, and their combination on *B. rapa* germination success and development. We focused on these two flavones, as the concentration of their glycosidic derivatives was reported to be low during the fall [47]. The chemical properties and the solubility of both API and LUT have been amply characterized in a variety of solvents, including water [147,148,149,150]. The water solubility of API is as low as 183 µg mL^−1^ [147,148], while LUT’s is 387.5 µg mL^−1^ at 25 °C [149,150]. Compared to their solubility in other solvents, API and LUT’s solubility in water is low [147,148,149,150]. The poor solubility of these substances implies that the bioactive concentrations of API and LUT cannot exceed 183 µg mL^−1^ and 387.5 µg mL^−1^, respectively, under field conditions and in the soil. These values also reflect the likely maximum extractable amounts of API and LUT from leaves in an aqueous extract, under ideal conditions. However, under field conditions, it is likely that the water-soluble API and LUT amounts released from degrading leaves are significantly lower, considering that leaf degradation occurs at relatively low temperatures in the fall, which may further decrease API and LUT solubility in the aqueous environment [147,148,149,150]. Thus, they are likely to be present in fully aqueous extracts at low concentrations compared to the amount extractable using organic solvents, in which their solubility is higher [47,146]. However, these flavones can still accumulate in the soil at bioactive concentrations that are relevant for allelopathic interactions [102].

In this study, we treated standard *B. rapa* seeds with 50 µg mL^−1^ API, LUT, and a 1:1 ratio combination of the two. These concentrations are lower than those used in prior studies [47] and represent a concentration range that plants are more likely to be exposed to under field conditions. We found that the two metabolites have different effects on *B. rapa*. Surprisingly, API stimulated *B. rapa* seed germination. Other studies also observed similar stimulating effects triggered by different members of the phenylpropanoid family, the group of metabolites to which API belongs. For example, Bi et al. (2017) [151] documented that sinapic acid stimulates the germination of wild-type Arabidopsis seeds in a concentration-dependent manner in the range of 0.1 to 1 mM.

On the other hand, API significantly inhibited seedling growth. LUT repressed both germination and growth. The combination of apigenin and luteolin decreased germination to a similar extent as LUT alone. However, the combination had a much stronger effect on seedling growth than either of the two flavones alone, suggesting that the simultaneous occurrence of multiple metabolites in degrading leaves may result in enhanced allelopathic effects (Figure 6). In addition, stereomicroscopic examination revealed aberrant morphology and deficiency in the root development of seedlings treated with API and LUT combined (Figure 7).

From the obtained data, we conclude that the net allelopathic impacts of degrading *L. maackii* leaves on *B. rapa* are controlled by complex chemistry and the combined effects of multiple metabolites. In particular, the described effects of aqueous *L. maackii* extracts are similar to those triggered by the combination of API and LUT (Figure 3, Figure 4, Figure 5, Figure 6 and Figure 7), supporting earlier assumptions made by Cipollini and colleagues [47]. Nevertheless, we do not exclude the possibility of different metabolites also playing a role in the net allelopathic effects of *L. maackii*, on which our study did not focus.

To better understand the mechanisms at the basis of our observations, future work will test for GA_3_ recovery effects on the allelopathic inhibition by API, LUT, and their combination, will test for potential synergistic or antagonistic effects of the API + LUT combination, and will be complemented with a comparative analysis of responses to API, LUT and API + LUT of the varieties differing in their ability to synthesize GA_3_.

In conclusion, this study demonstrated that the net allelopathic potential of *L. maackii* leaves is defined by complex chemistry and the interactive effects of multiple metabolites on the target species. For the first time, we showed that the target’s metabolic properties might also influence the net allelopathic impacts of invasives, in addition to environmental factors and the proximity to the source. In particular, high GA_3_ concentrations may substantially alleviate the inhibitory effects of *L. maackii* allelochemicals. The mechanism responsible for this effect will be further investigated in the future. A better understanding of the direct impacts of allelochemicals on target species will contribute to developing novel invasive species control and biodiversity conservation protocols. In addition, this knowledge may also contribute to additional applications in other fields—for example, to support applications employing allelopathic species in agriculture [11,89,92,152,153,154], in water recycling [155], and as substitutes for synthetic herbicides [136,156].

## 4. Materials and Methods

### 4.1. Plant Material and Growth Conditions

#### 4.1.1. *Lonicera maackii* Leaf Collection and Extraction

*L. maackii* (Amur honeysuckle) leaves were collected from honeysuckle-infested forest stands in the Otoe Creek Natural Area on the Missouri Western State University campus (GPS coordinates: 39.7597° N, 94.7845° W) on the third week of each month between March and November 2020 and in 2021, throughout the growing season. Species identification was provided by the Missouri Department of Conservation’s Northwest Regional Office, located on the Missouri Western State University campus. The harvested leaves were rinsed three times with sterile distilled water to remove surface contaminants, blotted, frozen, and stored at −20 °C in a freezer (Frigidaire, Charlotte, NC, USA) until processing.

Aqueous extracts were prepared from whole leaves, modifying a method previously used by Dorning and Cipollini [51]. The leaves were incubated and shaken without disruption in sterile distilled water at a concentration of 2 g (leaf tissue) mL^−1^ at room temperature for 72 h. The extract stock was decanted and filtered using a Pall Acrodisc^®^ sterile syringe filter with Supor^®^ membrane, with a 0.2 µm pore-size and 32 mm OD (Pall Corp., Port Washington, NY, USA). The filtered extracts were stored at 4 °C until use in a walk-in cooler unit (Amerikooler QC061277FBSC, Hialeah, FL, USA).

For germination and seedling development tests, the stock extract of 2 g (leaf tissue) mL^−1^ was diluted with sterile distilled water to obtain concentrations of 0; 0.5; 0.75; 1; 1.5; and 2 g (leaf tissue) mL^−1^.

#### 4.1.2. *Brassica rapa* Varieties and Growth Conditions

The effects of *L. maackii* leaf extracts and their chemical components on seed germination and seedling growth were tested on Wisconsin Fast Plants^®^ *Brassica rapa* varieties in Petri dish seed assays. Wisconsin Fast Plants^®^ *Brassica rapa* subsp. *dichotoma* [80] seed stocks were obtained from the University of Wisconsin-Madison, WI, USA and stored at 4 °C until testing in a walk-in cooler.

Standard Wisconsin Fast Plants^®^ seeds (*Rbr*) were used as controls. The Standard stock characterizes the typical morphology and growth patterns for Fast Plants^®^ [81,82].

The Rosette-Dwarf variety (*ros*) is a Fast Plants^®^ stock homozygous recessive (*ros*/*ros*) for a gene that causes deficiency in the synthesis of the growth hormone gibberellic acid (GA_3_). Dwarf plants produce 4–10 times less GA_3_ than Standard (*Rbr*) plants, resulting in stems that do not elongate after seedlings emerge [81,82]. 

The Tall Plant (Elongated Internode, *ein*) variety is a Fast Plants^®^ stock homozygous recessive (*ein/ein*) for a gene causing the over-production of phytohormones in the gibberellin family, resulting in overly elongated stems and internodes. Tall plants produce up to 12 times more GA_3_ than Standard (*Rbr*) Wisconsin Fast Plants^®^. This stock develops tall plants [81,82].

For each seed assay, 20 *Brassica rapa* seeds of either variety were placed on wet filter paper in 15 × 100 mm plastic Petri dishes. A 5 mL solution of the selected composition and concentration was added to each Petri dish. The plates were sealed with parafilm to minimize evaporation. The dishes were placed on racks of a climate-controlled growth chamber (Percival, AR36L2, Perry, IA, USA). The plated seeds were kept under a day/night temperature cycle of 27/25 °C and a cycle of 16 h days with 250 µmol m^−2^ s^−1^ intensity irradiation and 8 h of dark for the duration of each assay.

Three independent replicates (N = 3) were performed for each treatment and seed variety for all assays, with 60 individual seeds of either type examined under each condition (n = 60).

### 4.2. Germination and Seedling Development Bioassays

#### 4.2.1. Concentration Dependence and Seasonal Variation in the Effects of *Lonicera maackii* Extracts on Brassica Seeds

The concentration dependence of the effects of *L. maackii* leaf extracts on Standard (*Rbr*) control seeds was tested within the 0–2 g (leaf tissue) mL^−1^ concentration range. The extracts were prepared from the stock harvested in October 2020. *Rbr* seed germination rates were recorded at each applied concentration as the percent fraction of water-treated control seeds after 24 h.

Seasonal variations in the inhibitory potential of honeysuckle extracts prepared from leaves harvested throughout the 2020 and 2021 growth seasons, from March to November, were assessed in Petri dish bioassays. Such variations were expected to serve as a proxy for the dynamic synthesis and accumulation of inhibitors in the leaf tissue throughout the plant’s life cycle.

*Rbr* seeds were treated for 24 h with 1 g (leaf tissue) mL^−1^ extracts of leaves harvested on the third week of each month during the honeysuckle life cycle, combining leaves harvested in the same months of 2020 and 2021. The choice of the extract concentration for this assay was based on preliminary data indicating that, at this concentration, extracts prepared from leaves harvested in October may inhibit seed germination by over 80%, but no lethal effects have been observed in *Rbr* seeds yet.

#### 4.2.2. The Impact of the Plant Growth Hormones, Gibberellins, on Brassica Seed Responses to *Lonicera maackii* Leaf Extracts

The Standard (*Rbr*), Rosette-Dwarf (*ros*), and Tall (*ein*) *Brassica rapa* Fast Plant^®^ varieties differ in their endogenous synthesis and accumulation of gibberellins [80,81,82]. To compare their sensitivity to the *L. maackii* leaf extracts, the seeds of each variety were treated with 1 g (leaf tissue) mL^−1^ *L. maackii* extract for 72 h in Petri dishes, in parallel with the water-treated controls for each variety.

For assessing the potential impact of exogenous gibberellin supplementation on Brassica seed responses, Petri dishes with *Rbr*, *ros*, and *ein* seeds, treated with *L. maackii* extract, were also supplemented with 100 µM sterile aqueous solution of GA_3_ (Millipore Sigma, Merck KgaA, Darmstadt, Germany). Each seed variety’s control was treated with sterile distilled water instead of the *L. maackii* extract.

Germination and seedling growth were measured at 24 h intervals for up to 72 h. Germination rates were expressed as a percent (%) fraction of the water-treated controls’ germination under each condition for each variety. The seedling length was measured in centimeters (cm).

#### 4.2.3. Apigenin, Luteolin, and Their Combined Effects on Brassica Seed Germination and Early Seedling Development

To assess the individual or combined effects of these flavones on the germination and early-stage development of *Rbr* Brassica seedlings, seeds were treated with 50 µg mL^−1^ solutions of API, LUT, and the combination of 50 µg mL^−1^ API + 50 µg mL^−1^ LUT in 0.05% DMSO (Millipore Sigma, Merck KgaA, Darmstadt, Germany). The seed response to the flavones was assayed in Petri dishes, with the treatments lasting up to 48 h. Control seeds were treated with sterile distilled water. Germination rates were expressed as the % change over time, and seedling lengths were established as the average lengths of all germinated seedlings at the time of the measurements for each variety and treatment. Germination and seedling development were assessed at a frequency of 12 h for the 48 h duration of the test.

Representative images of the developing control and treated (API, LUT, or API + LUT combination) *Rbr* Brassica seedlings were photographed under the lowest magnifying power (1.5×) of a Labomed Luxeo 4Z Stereomicroscope (Labo America Inc., Fremont, CA, USA) fitted with an AmScope CA-CAN-SLRIII adapter (United Scope, Llc., Los Angeles, CA, USA) for a Canon EOS Rebel T7 DSLR Camera (Canon Inc., Melville, NY, USA).

### 4.3. Statistical Analysis

All statistical analyses were performed using the Statistical Package for the Social Sciences software, version 20 (SPSS Inc., Chicago, IL, USA). Data were expressed as the means ± standard deviation (SD) of three independent replicates (N = 3, n = 60 seeds total for each treatment and condition). Student’s *t*-test was used to compare the differences in Brassica seed responses to varying honeysuckle extract concentrations and to analyze the seasonal variation in the *L. maackii* extracts’ negative allelopathic efficiency. Students’ test was also used to assess the significance of different variety seed germination and growth changes in response to *L. maackii*-induced inhibition in the presence and absence of exogenous GA_3_ compared to the corresponding controls. The same test was used to determine the significance of the impact of flavones or their combination on *Rbr* seed germination and seedling growth after 48 h, as compared to the controls. Analysis of variance (two-way ANOVA) was used to test for differences in the germination and growth of the used *B. rapa* varieties treated with *L. maackii* extracts without or in the presence of exogenous GA_3_ over time. Graphing was performed with Microsoft^®^ Excel^®^ 365 MSO, Version 2210 (Microsoft Corporation, Redmond, WA, USA).

## Data Availability

Data can be made available upon request.
